# Using TOF-SIMS Spectrometry to Study the Kinetics of the Interfacial Retro Diels–Alder Reaction

**DOI:** 10.3390/ma14102674

**Published:** 2021-05-20

**Authors:** Lilia Hassouna, Sachin Kumar Enganati, Florence Bally-Le Gall, Grégory Mertz, Jérôme Bour, David Ruch, Vincent Roucoules

**Affiliations:** 1 Materials and Research Technology Department, Luxembourg Institute of Science and Technology, 5 Avenue des Hauts-Fourneaux, L-4362 Esch-sur-Alzette, Luxembourg; hassounalilia@gmail.com (L.H.); sachinkumar.enganati@list.lu (S.K.E.); jerome.bour@list.lu (J.B.); david.ruch@list.lu (D.R.); 2 Department of Physics and Materials Science, University of Luxembourg, 2 Avenue de l’Université, L-4365 Esch-sur-Alzette, Luxembourg; 3 University of Haute-Alsace, University of Strasbourg, CNRS, IS2M UMR 7361, F-68100 Mulhouse, France; florence.bally-le-gall@uha.fr (F.B.-L.G.); vincent.roucoules@uha.fr (V.R.)

**Keywords:** interfacial reaction, retro Diels–Alder, kinetics, TOF-SIMS

## Abstract

In this work, the use of Time of Flight Secondary Ion Mass Spectrometry (TOF-SIMS) was explored as a technique for monitoring the interfacial retro Diels–Alder (retro DA) reaction occurring on well-controlled self-assembled monolayers (SAMs). A molecule containing a Diels–Alder (DA) adduct was grafted on to the monolayers, then the surface was heated at different temperatures to follow the reaction conversion. A TOF-SIMS analysis of the surface allowed the detection of a fragment from the molecule, which is released from the surface when retro DA reaction occurs. Hence, by monitoring the decay of this fragment’s peak integral, the reaction conversion could be determined in function of the time and for different temperatures. The viability of this method was then discussed in comparison with the results obtained by ^1^H NMR spectroscopy.

## 1. Introduction

The well-known Diels–Alder (DA) and retro Diels–Alder (retro DA) chemistry has been widely used in the design of stimuli-responsive interfaces. Examples concern mainly applications in adhesion [1,2], and in the controlled immobilization and release of molecules [3,4]. The unique thermoreversible character of this reaction allows the formation (Diels–Alder reaction) and dissociation (retro Diels–Alder reaction) of an adduct with a simple change of temperature and without any side reactions [5]. However, despite the importance of these reactions, the study of kinetics and thermodynamics of DA reactions occurring at interfaces has only been reported in a few contributions [6,7].

In general, studies of reactions that occur at the solid–liquid interface lag far behind studies of reactions in solution [8]. This can be explained by the difficulty in finding appropriate analytical techniques able to probe and distinguish reactants from products at the molecular scale in confined and complex environments. A few analytical techniques were proven efficient in monitoring interfacial reactions, they consist mainly of advanced water contact angle [9,10] and cyclic voltammetry [11,12,13] measurements.

Hence, it is necessary to broaden the arsenal of analytical techniques that allow an efficient investigation of interfacial reactions especially in cases where the density of the analyte at the studied surfaces is low, and where the aforementioned techniques are not sensitive enough to allow the detection of the desired compounds. TOF-SIMS is one of the most promising candidates for high-sensitivity surface analysis: it has low detection limits and gives molecular and chemical information on the surfaces of different materials [14]. However, the data quantification in TOF-SIMS is not straightforward because of complex matrix effects, necessitating either the development of calibration curves [15,16] or its use in conjunction with another analytical technique such as XPS [17], surface plasmon resonance (SPR) [18] or UV–visible spectroscopy [19].

In this work, TOF-SIMS is evaluated as a direct method to monitor an interfacial reaction [20] that occurs on well-controlled and reproducible SAMs [21,22]. The results obtained were assessed in comparison with the results obtained from a well-established quantitative technique: ^1^H NMR spectroscopy.

## 2. Materials and Methods

Chemicals: 1,1′methylenedi-4,1-phenylene-bismaleimmide (95%), furfuryl glycidyl ether (96%), acetonitrile (99.5%), n-heptane (99%), hydrochloric acid (37%) triethylamine (99.5%), dimethyl sulfoxide-d6 (99.5 atom % D) were purchased from Sigma Aldrich (Diegem, Belgium). Ethanol absolute (99%), dimethylformamide (99.8%), sodium azide (99%), lithium aluminium hydride (1 M in THF), dimethyl sulfoxide (>99.7%) were obtained from Acros Organics and bromo-undecyltrichlorosilane (95%) from abcr (abcr GmBH, Karlsruhe, Germany). Silicon wafers <100> type were purchased from Si-Mat. Tetrahydrofuran and chloroform (SLR grade, Thermo Fisher (Waltham, MA, USA) were purified through an MBraun SPS solvent purification system.

DA adduct synthesis was carried out according to a method by Min et al. [23]. Briefly, 0.014 mol of 1,1′methylenedi-4,1-phenylene-bismaleimmide (BMI) were dissolved in THF before adding 0.028 mol of Furfuryl Glycidyl ether (FGE). The solution was then refluxed at 70 °C for 24 h under a nitrogen atmosphere. The final product was obtained after purification on silica gel.

Matrix-assisted laser desorption/ionization high-resolution mass spectrometry (MALDI-HRMS) analyses: Measurements were performed on an AP/MALDI UHR (ng) source (MassTech Inc., Columbia, MD, USA) employing a Nd:YAG laser at 355 nm wavelength coupled to an LTQ/Orbitrap Elite mass spectrometer (Thermo Fisher Scientific, Bremen, Germany). α-cyano-4-hydroxycinnamic acid (CHCA) was selected as the matrix.

SAMs preparation [22]: The deposition of amine-terminated SAMs was achieved through three steps involving the immersion of the substrates (previously cleaned with piranha solution) into a solution of the adequate reagent. First, bromo-undecyltrichlorosilane was deposited on silicon wafers by immersing the substrates in a diluted solution of the silane. Then, bromine groups were transformed into azide groups by reaction with NaN_3_ via an S_N_2 nucleophilic substitution. Finally, immersion in an LiAlH_4_ solution allowed the reduction of azide groups into amine groups. The immersion of these surfaces in a DA linker solution (2 × 10^−2^ M) in a 2:1 mixture of ethanol and THF allowed the grafting of the adduct on to the surface.

Interfacial retro DA monitoring protocol: Surfaces that contain the DA adduct were immersed in fresh dimethyl sulfoxide (DMSO) at various temperatures, then samples were collected at different reaction times. The substrates were thoroughly rinsed before analysis with TOF-SIMS.

TOF-SIMS analyses: Measurements were performed in positive ion mode with a commercial TOF-SIMS V (IonTOF GmbH, Münster, Germany) instrument. The analyses were carried out using a pulsed bismuth liquid metal ion gun (LMIG, Bi^3+^ ions, 25 keV) delivering 0.40 pA target current. The area analysed was 500 µm × 500 µm. The analyses were performed using a primary ion dose density maintained to 10^11^ ions/cm^2^.The data acquisition and processing software was Surface Lab 7.0 (Version 7.0.106074, ION-TOF GmbH, Münster, Germany). Five different areas were analysed on each surface. To limit the well-known matrix effect, all samples have been prepared in the same manner and followed the same treatment during the kinetic meaning that each surface present the same environment. Then, the intensity of the peak of interest for each resulting spectrum was individually normalized by the Total Ion Current (TIC) to avoid any signal variation during the analysis on the sample surface.

Retro DA reaction in solution: The reaction was followed by ^1^H NMR spectroscopy. The temperature of the tube was changed in situ and spectra were taken at different reaction times. NMR spectra were recorded on a Bruker AVANCE III HD 600 spectrometer (Billerica, MA, USA) (600 MHz). 5.0 mm multi-nuclear observe probe with z-gradient.

## 3. Results and Discussions

The synthesized DA molecule was first well characterized by Nuclear Magnetic Resonance (NMR) spectroscopy (Figure S1), with the results being in accordance with other studies [23]. MALDI-HRMS mass spectrometry allowed the different fragments resulting from the molecule to be detected, as depicted in Figure S2 and Table S1, which confirmed the structure of the molecule.

From the other hand, well controlled amine terminated SAMs (called hereafter SAMs-NH_2_) were prepared by deposition of long silane chains terminated with non-nucleophilic groups according to the work by Böhmler et al. [22]. XPS characterization of these surfaces can be found in the supporting information Figure S3.

**Figure 1:**
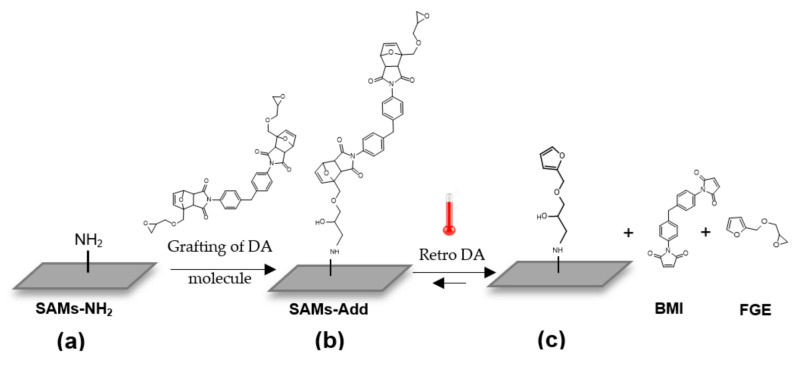
Scheme representing amine-terminated SAMs (**a**) on to which a molecule containing a DA adduct was grafted to give surface (**b**). Retro DA reaction occurred on the adduct to give a furan terminated surface (**c**) liberating one BMI and FGE molecules.

The DA adduct was then grafted on to amine-terminated SAMs (SAMs-NH_2_) to give the surfaces SAMs-Add (as shown in Figure 1 going from surface (a) to (b)).

These surfaces were then analysed using TOF-SIMS spectrometry in positive ion mode. It is known that ion bombardment in TOF-SIMS generates extensive molecular fragmentation therefore no peaks corresponding to the mass of the whole DA adduct, the bismaleimide (BMI) or the Furfuryl Glycidyl ether (FGE) were detected.

**Figure 2:**
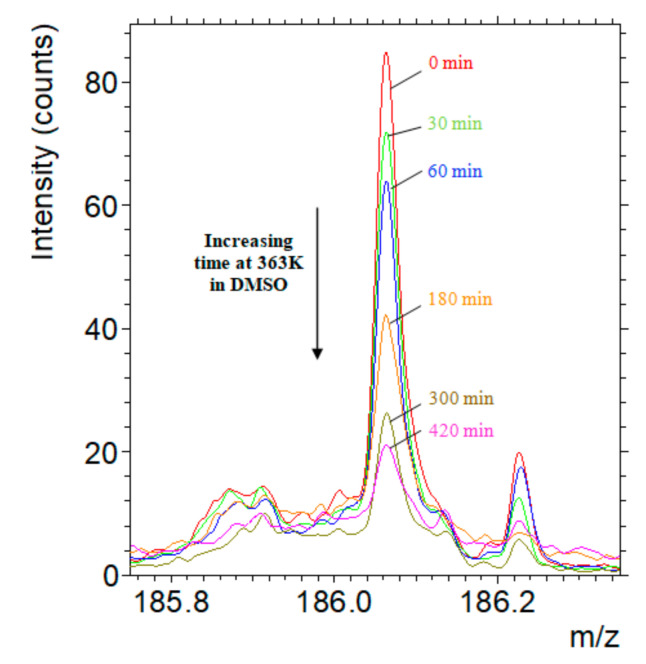
Decrease of the peak area representing the maleimide-containing fragment as SAMs-Add surface is heated up to 363 K for 0 min, 30 min, 1 h, 3 h, 5 h, and 7 h.

However, one peak was identified on SAMs-Add that was not present on SAMs-NH_2_ (Figure 2). This peak has an experimental mass of *m*/*z* = 186.0577 and corresponds to a BMI fragment also detected when the DA molecule was analysed in powder form by MALDI-HRMS (molecular ion N°1 in Table S1 with the chemical formula: C_11_H_8_O_2_N^+^). Therefore, this fragment came from the DA molecule grafted on to the surface. The theoretical mass of the fragment is *m*/*z* = 186.0555 leading to a mass error of 11.8 ppm.

This fragment was still detectable on the SAMs-Add surface even after cleaning it in an ultrasonic bath, confirming that the adduct molecule was covalently attached to the surface [24].

### 3.1. Investigation of Interfacial Retro DA Reaction Using TOF-SIMS

SAMs-Add surfaces were used as a starting point for the study of interfacial retro DA reactions. They were heated in DMSO at different temperatures for different times to follow the evolution of the reaction conversion.

When retro DA reaction occurs on SAMs-Add, the bismaleimide is released from the surface and the anchored molecule becomes furan-terminated (Figure 1c). Hence, the integral of the peak representing the fragment containing maleimide is expected to decrease as the reaction progresses. A preliminary experiment was conducted to validate this hypothesis. SAMs-Add surfaces were introduced in DMSO at high temperature (363 K), and samples were collected at different time intervals. After an appropriate washing and drying of the surface, it was analysed by TOF-SIMS and compared with the initial surface, which did not undergo retro DA reaction. Figure 2 presents the evolution of the peak characteristic of the maleimide-containing fragment with the time of immersion in DMSO at 363 K.

The results showed that the integral of the peak corresponding to the maleimide-containing fragment (*m*/*z* = 186.0577) decreased when SAMs-Add surfaces were heated up in DMSO and this decrease was more important with time. This is a clear indication that the maleimide was released continuously from the surface via a reaction that is dependent upon temperature, i.e., retro DA reaction (see Figure 1 going from surface b to c). Consequently, by following the decay of the maleimide-containing fragment density on the surface with time, the retro DA reaction can be monitored.

When retro DA reaction occurs, the anchored DA molecule becomes terminated with a furan (Figure 1); hence, the reaction conversion can be defined as the fraction of furan groups that are present on the surface: X_furan_. The remaining groups on the surface are in adduct form, their fraction is referred to as X_Add_ (X_Add_ will decrease with time as the adduct is consumed by retro DA reaction while X_furan_ will increase). This means that at each time:

X_Add_ + X_furan_ = 1 (1)

The retro DA reaction conversion is then equivalent to the furan density on the surface; it is calculated as follows:

Conversion: X_furan_ = (A_0_ −A_t_)/A_0_ (2)

A_0_ is the integral of the peak of the maleimide fragment (*m*/*z* = 186.0577) before the retro DA reaction. It gives us a reference for the total adduct content of the surface. A_t_ is the integral of that same peak at a given time. Integrals are normalized to the Total Ion Current (TIC). The values of the integrals used in this calculation can be found in Table S2.

**Figure 3:**
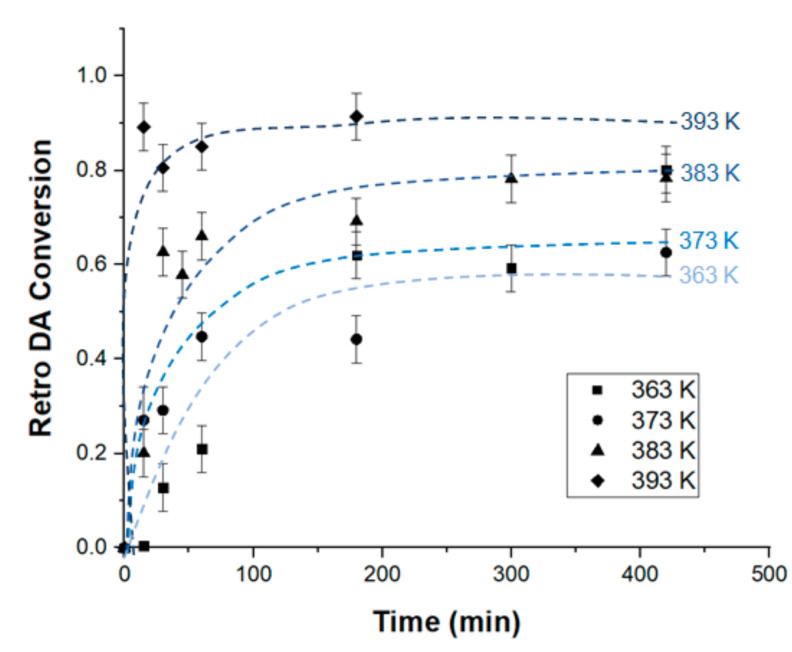
Evolution of retro DA reaction conversion on SAMs with time, at different temperatures. The lines were drawn only to guide the eyes of the reader.

Conversion values obtained for different temperatures are presented in Figure 3. It is clear that the reaction conversion increases with the increase of the temperature of the medium. The DA reaction exists in a dynamic state, the equilibrium of which can be shifted from a dominating adduct formation to a predominant retro DA reaction depending on the temperature. At the selected temperatures: 363, 373, 383, and 393 K, retro DA reaction is considered predominant and DA reaction can be neglected [25].

The first order rate law of retro DA reaction can be written as follows:

ln χ_Add_ = − k_rDA_ t + ln χ_Add(t = 0)_ (3)

k_rDA_ is the retro DA rate constant, χ_Add_ is the surface content of the DA adduct and χ_Add(t = 0)_ is the surface fraction of the DA adduct before retro DA reaction (t = 0).

**Figure 4:**
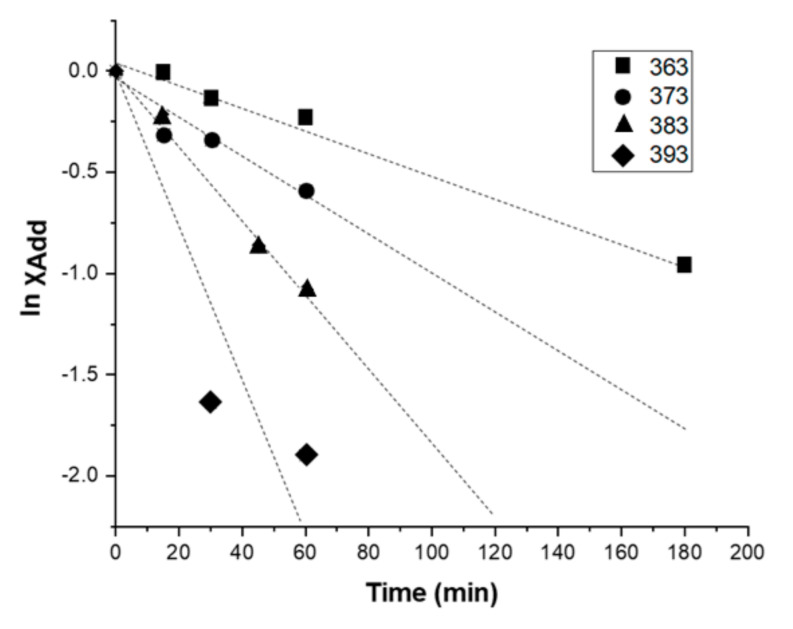
Linearization according to a first order of retro DA reaction of the adduct performed at different temperatures.

χ_Add_ values were determined from Equations (1) and (2) at each time for each temperature, which allowed us to plot (ln χ_Add_ versus time) in Figure 4. The linear fitting of the plots gives R^2^ correlation coefficients of 0.99; 0.90; 0.99, and 0.85, respectively, for temperatures of 363, 373, 383, and 393 K. The linearity of these plots confirms that the reaction is of first order.

Equation (3) implies that the rate constant of retro DA reaction can be determined at each temperature directly from the slopes of the plots ln χ_Add_ = f(time). The values of the rate constants are presented in Table 1.

Knowing the values of rate constants at different temperatures, the value of retro DA reaction activation energy (E_a_) can be estimated thanks to the linear form of Arrhenius law:

ln k_rDA_ = ln A − E_a_/RT (4)

**Figure 5:**
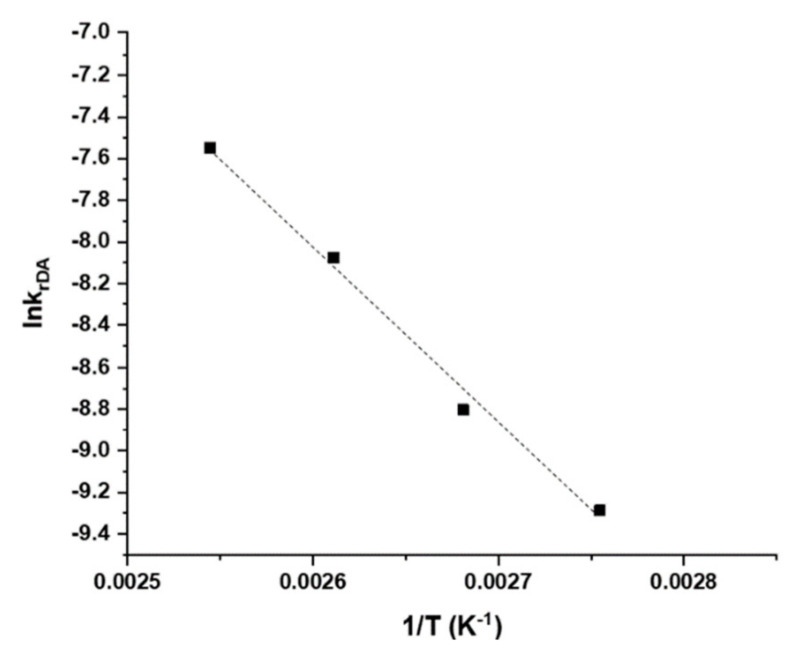
Determination of interfacial retro DA activation energy by linearization of the Arrhenius equation: ln k_rDA_ = −8472.2 × 1/T + 14.004; R^2^ = 0.99.

A is the pre-exponential factor, a constant for each chemical reaction. R is the universal gas constant and T the absolute temperature.

ln k_rDA_ = f (1/T) is plotted in Figure 5. The fitting equation: ln k_rDA_ = −8472.2/T + 14.004 allowed an estimation of the activation energy E_a_ = 72 ± 3 kJ·mol^−1^, from the slope of the plot. The standard errors in the calculation of activation energy were obtained from the linear regressions.

The activation energy value is in the same order of magnitude as the activation energy of an interfacial retro DA reaction between a furan and maleimide, as determined by computational calculations in another work: (96 kJ·mol^−1^) [26]. This result, along with the linear fitting of the conversion values to a first order reaction, are a first indication that the results obtained with TOF-SIMS are reliable and that matrix effects can be neglected in this case. In the next section, the activation energy of this reaction will be determined in solution by ^1^H NMR spectroscopy to support this hypothesis.

### 3.2. Investigation of Retro DA Reaction Using ^1^H NMR Spectroscopy

**Figure 6:**
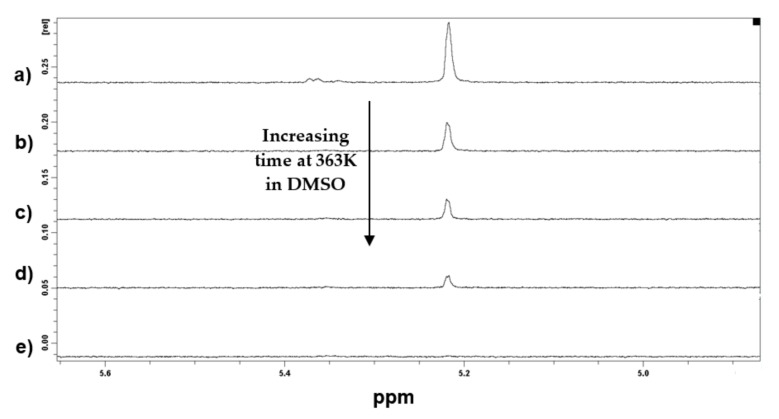
Decrease of the adduct peaks at 5.35 ppm and 5.20 ppm in ^1^H NMR spectra of DA-containing molecule performed in deuterated DMSO at 363 K after (**a**) 0 min, (**b**) 15 min, (**c**) 30 min, (**d**) 45 min, and (**e**) 120 min.

NMR spectroscopy was also used to study the same reaction in solution (using the same solvent: DMSO) by following a peak representing one proton characteristic of the furan–maleimide adduct. This peak is well documented in the literature [27] and consists of two components each one belonging to a stereoisomer of the adduct: δ_endo_ = 5.35 ppm and δ_exo_ = 5.20 ppm. Figure 6 shows the decrease of these peaks with time at 363 K.

The disappearance of one adduct implies the formation of one furan and one maleimide group. Hence, by following the integral of a peak representing one proton of the furan, it was possible to calculate the reaction conversion. The ^1^H NMR spectra of all components are presented in Figure S4. More details on the methodology used for the determination of reaction conversion can be found in the supporting information, and the values of the reaction conversion determined at four different temperatures can be found in Table S3.

Using the same methodology described above, (ln χ_Add_ versus time) was plotted (Figure S5). Similarly, the reaction rate constants (k_rDA(s)_) were determined from the slopes of these plots (Table 2).

**Figure 7:**
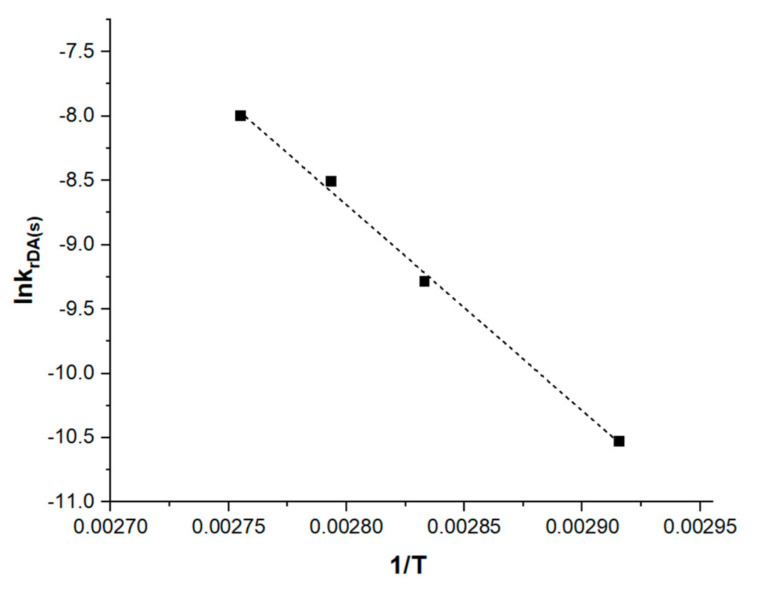
Determination of retro DA activation energy in solution by linearization of the Arrhenius law equation: ln k_rDA(s)_ = −15998 × 1/T + 36; R^2^ = 0.99.

Then, ln k_rDA(s)_ = f (1/T) was plotted for the four temperatures (Figure 7). After that, thanks to the Arrhenius law, activation energy was calculated from the slope of the plot as E_a_= 132 ± 2 kJ·mol^−1^. The standard errors in the calculation of activation energy were obtained from the linear regressions. This value is very close to the value found in literature [28] for retro DA reaction between furan and methyl maleimide occurring in DMSO (134 ± 4 kJ·mol^−1^).

### 3.3. Discussion of Activation Energy Results

The activation energy of retro DA reaction in solution (E_a_= 132 ± 2 kJ·mol^−1^) is in the same order of magnitude as the activation energy of the same reaction on SAMs (E_a_= 72 ± 3 kJ·mol^−1^), which is coherent since it is the same reaction that is observed in the same solvent (DMSO).

**Figure 8:**
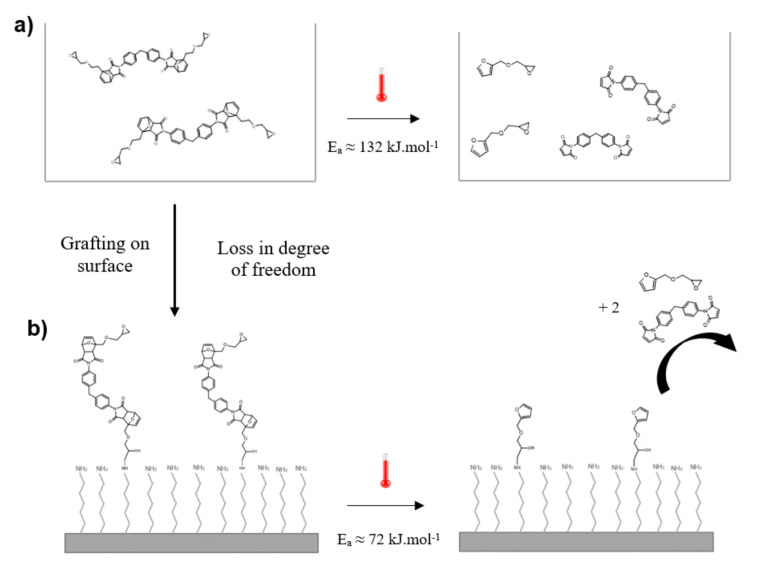
Schematic representation of retro DA reaction occurring (**a**) in solution and (**b**) on SAMs.

However, the molecules on SAMs are attached to the surface and lose one degree of freedom compared to the completely free molecules in solution (Figure 8). Since the molecules on SAMs are more stable, they can take fewer conformations, which facilitates the reaction. Indeed, the adduct must take a specific conformation forretro DA reaction to occur [29]. This explains the lower value of activation energy on SAMs then in solution where the molecules have more degrees of freedom and an important part of the thermal energy is dissipated in molecular vibrations.

^1^H NMR spectrometry is widely used as a quantitative technique for reaction monitoring and gives accurate and reliable values of kinetic parameters [30]. In this work, the activation energy of retro DA reaction determined by NMR validates the activation energy on SAMs determined using TOF-SIMS. This supports TOF-SIMS as a technique for reaction monitoring on the surface level, which enlarges the arsenal of techniques that allow interfacial reaction studies. Of course, matrix effects still exist [31], and for a more rigorous determination of the reaction activation parameters, it will be necessary in the future to develop calibration curves, for example by preparing standards with known surface densities.

## 4. Conclusions

In this work, TOF-SIMS was shown to be useful for the monitoring of retro DA reaction occurring at the solid–liquid interface, which allowed the determination of the activation energy using the Arrhenius law. The same methodology was then used to determine the activation energy of the same reaction occurring in solution using ^1^H NMR spectroscopy. This confirmed the results obtained by TOF-SIMS and the difference between the two values were discussed, it was concluded that retro DA reaction kinetics can change depending on the degree of freedom of the Diels–Alder adduct. This implies that the DA bonds cleavage can be controlled by changing the surface structure.

## Figures and Tables

**Table 1:** Retro DA rate constants k_rDA_ on SAMs determined for different temperatures.

Table (K)	k_rDA_ × 10^5^ (s^−1^)
363	9 ± 1
373	15 ± 3
383	31 ± 2
393	52 ± 10

**Table 2:** Retro DA rate constants k_rDA(s)_ in solution as determined by ^1^H NMR spectroscopy at different temperatures.

T (K)	k_rDA_ × 10^5^ (s^−1^)
343	2.7 ± 0.1
353	9.3 ± 0.5
358	20 ± 1
363	34 ± 2

## Data Availability

Data Sharing is not applicable.

## Supplementary Materials

The following are available online at https://www.mdpi.com/article/10.3390/ma14102674/s1, Figure S1: ^1^H NMR spectrum of the DA molecule in DMSO at 298 K, Figure S2: (a) Comparison between experimental and theoretical MALDI-HRMS spectra of the DA adduct-containing molecule (b) Fragments of the DA molecule peaks detected by MALDI-HRMS, Figure S3: High resolution XPS N1s spectrum of (a) azide terminated SAMs that were finally transformed to (b) amine-terminated SAMs, Figure S4: ^1^H NMR spectra of (a) furfuryl glycidyl ether, (b) bismaleimide, and (c) synthesized molecule containing the adduct, Figure S5: Linearization, according to a first order, of retro DA reaction rate law occurring in the DA molecule in solution performed at different temperatures, Table S1: Structural assignments for the main fragments of the adduct molecule detected by MALDI-HRMS, Table S2: Values of the maleimide-fragment peak area as determined by TOF-SIMS, on SAMs, Table S3: Retro DA conversion values determined by ^1^H NMR spectrometry, for different temperatures.
