# Supplementary material for: Using TOF-SIMS Spectrometry to Study the Kinetics of the Interfacial Retro Diels–Alder Reaction
Source: Materials (Basel). 2021 May 20;14(10):2674. doi: 10.3390/ma14102674 (PMC8161361; doi:10.3390/ma14102674)
Supplement: Supplementary file 1 [file materials-14-02674-s001.zip › materials-1181722-SI.pdf]

materials

Supplementary Information

Using TOF-SIMS Spectrometry to Study the Kinetics of the Interfacial Retro Diels–Alder Reaction

Lilia Hassouna [1,2], Sachin Kumar Enganati [1,2], Florence Bally-Le Gall [3], Grégory Mertz [1,*], Jérôme Bour [1], David Ruch [1] and Vincent Roucoules [3]

1 Materials and Research Technology Department, Luxembourg Institute of Science and Technology, 5 Avenue des Hauts-Fourneaux, L-4362 Esch-sur-Alzette, Luxembourg; hassounalilia@gmail.com (L.H.); sachinkumar.enganati@list.lu (S.K.E.); jerome.bour@list.lu (J.B.); david.ruch@list.lu (D.R.)
2 Department of Physics and Materials Science, University of Luxembourg, 2 avenue de l'Université, L-4365 Esch-sur-Alzette, Luxembourg
3 University of Haute-Alsace, University of Strasbourg, CNRS, IS2M UMR 7361, F-68100 Mulhouse, France; florence.bally-le-gall@uha.fr (F.B.-L.G.); vincent.roucoules@uha.fr (V.R.)
* Correspondence: gregory.mertz@list.lu

Citation: Hassouna, L.; Enganati, S.K.; Bally-Le Gall, F.; Mertz, G.; Bour, J.; Ruch, D.; Roucoules, V. Using TOF-SIMS Spectrometry to Study the Kinetics of the Interfacial Retro Diels–Alder Reaction. *Materials* **2021**, *14*, 2674. https://doi.org/10.3390/ ma14102674

Academic Editor: Grzegorz Mlostoń

Received: 29 March 2021
Accepted: 7 May 2021
Published: 20 May 2021

Publisher's Note: MDPI stays neutral with regard to jurisdictional claims in published maps and institutional affiliations.

Copyright: © 2021 by the authors. Submitted for possible open access publication under the terms and conditions of the Creative Commons Attribution (CC BY) license (http://creativecommons.org/licenses/by/4.0/).

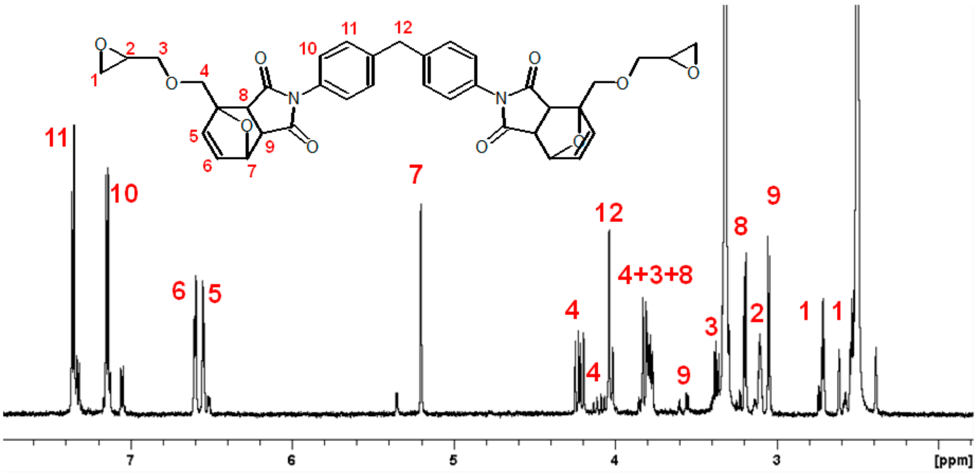

Figure S1. ^{1}H NMR spectrum of the DA molecule in DMSO at 298K.

Table S1. Structural assignments for the main fragments of the adduct molecule detected by MALDI-HRMS.

N°	Exact Mass	Observed Mass	Chemical Formula	Secondary Ion Structure
**1**	186.0555	186.0551	$C_{11}H_8O_2N^+$	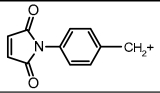
**2**	381.0846	381.0845	$C_{21}H_{14}O_4N_2Na^+$	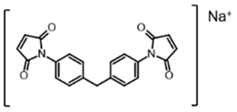
**3**	535.1481	535.1475	$C_{29}H_{24}O_7N_2Na^+$	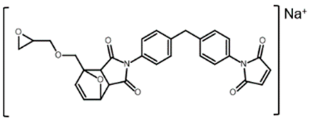
**4**	689.2106	689.2108	$C_{37}H_{34}O_{10}N_2Na^+$	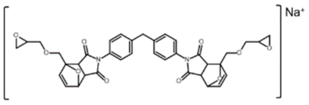

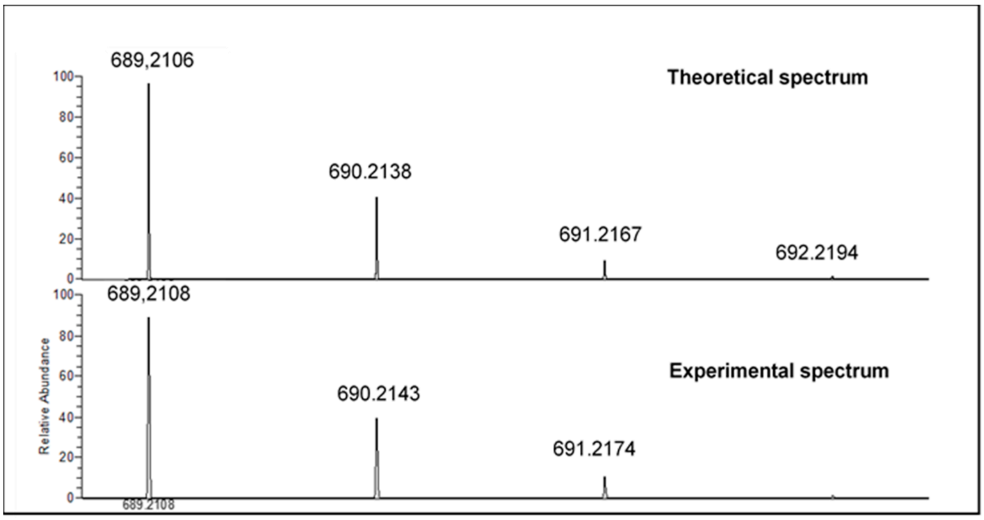

(**a**)

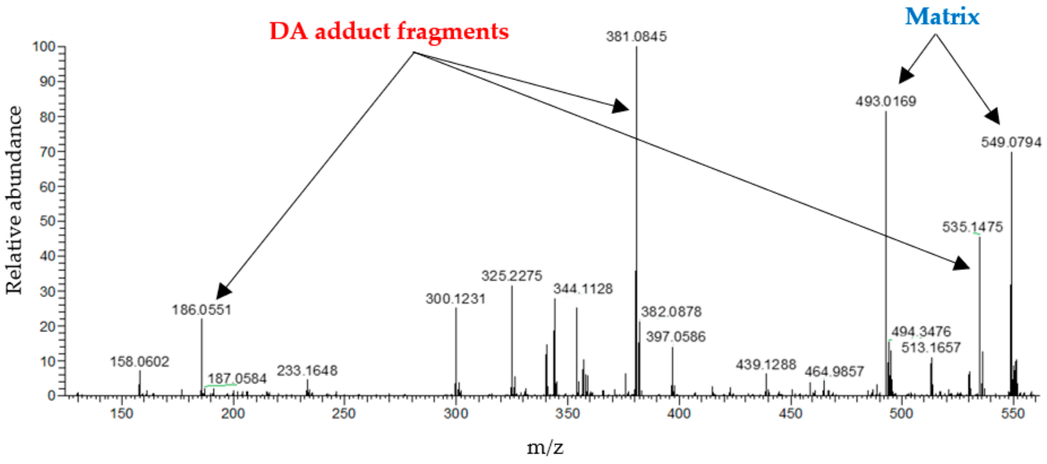

(**b**)

Figure S2. (**a**) Comparison between experimental and theoretical MALDI-HRMS spectra of the DA adduct-containing molecule (**b**) Fragments of the DA molecule peaks detected by MALDI-HRMS.

Table S2. Values of the maleimide-fragment peak area as determined by TOF-SIMS, on SAMs.

Time (s)	333 K					353 K				
**0**	0.0655	0.0601	0.0554	0.0352	0.0531	0.0099	0.0104	0.0109	0.0104	0.0102
**900**	-	-	-	-	-	0.0099	0.0094	0.0070	0.0103	0.0105
**1,800**	0.0591	0.0538	0.0627	0.0557	0.0610	0.0091	0.0082	0.0093	0.0072	0.0076
**3,600**	0.0312	0.0313	0.0273	0.0305	0.0327	0.0086	0.0076	0.0072	0.0069	0.0061
**10,800**	0.0126	0.0116	0.0115	0.0125	0.0127	0.0038	0.0039	0.0037	0.0037	0.0040
**Time (s)**	**363 K**					**373 K**				
**0**	0.0253	0.0160	0.0142	0.0142	0.0164	0.0099	0.0104	0.0109	0.0104	0.0102
**900**	0.0170	0.0173	0.0166	0.0176	0.0173	0.0079	0.0078	0.0062	0.0078	0.0083
**1,800**	0.0144	0.0143	0.0154	0.0156	0.0154	0.0068	0.0081	0.0071	0.0069	0.0078
**3,600**	0.0131	0.0133	0.0134	0.0148	0.0135	0.0054	0.0057	0.0057	0.0060	0.0059
**10,800**	0.0069	0.0064	0.0062	0.0067	0.0066	0.0057	0.0060	0.0055	0.0059	0.0058
**Time (s)**	**383 K**					**393 K**				
**0**	0.0253	0.0160	0.0142	0.0142	0.0164	0.0655	0.0601	0.0554	0.0352	0.0531
**900**	0.0142	0.0142	0.0138	0.0133	0.0134	-	-	-	-	-
**1,800**	-	-	-	-	-	0.0104	0.0104	0.0102	0.0105	0.0110
**2,700**	0.0078	0.0066	0.0065	0.0077	0.0077	-	-	-	-	-
**3,600**	0.0058	0.0062	0.0059	0.0057	0.0057	0.0094	0.0079	0.0082	0.0067	0.0083

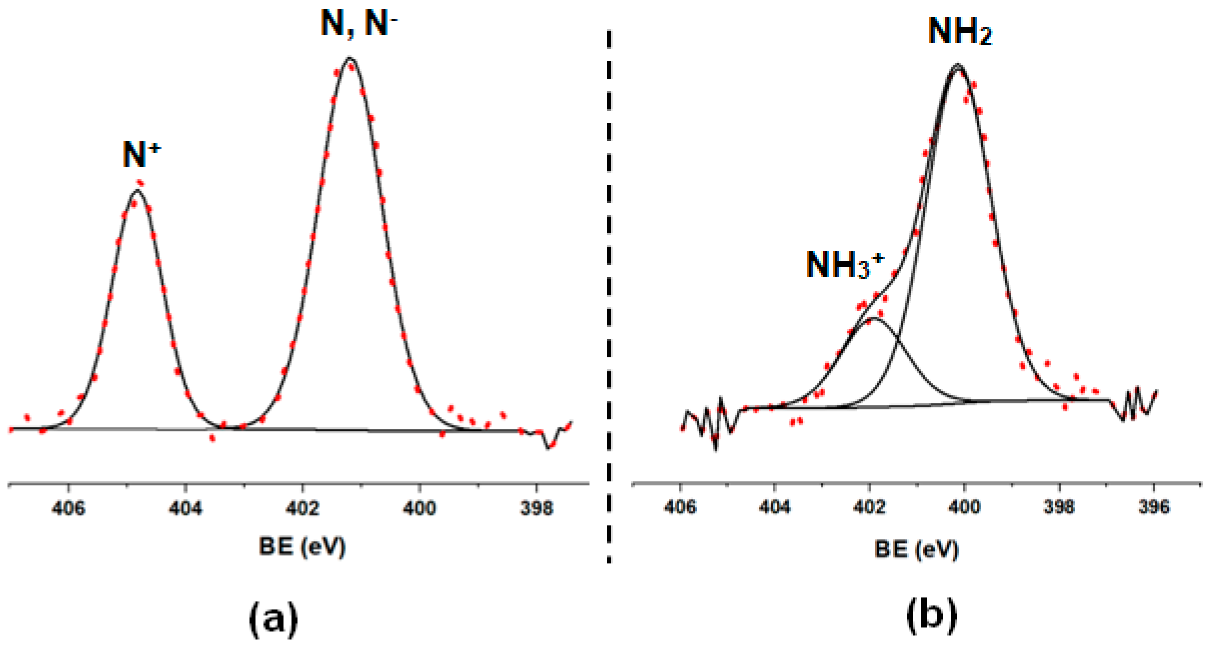

Figure S3. High resolution XPS N1s spectrum of (**a**) azide terminated SAMs that were finally transformed to (**b**) amine-terminated SAMs.

Methodology used for the determination of retro DA reaction conversion using ^1H NMR.

It is assumed that at the selected temperatures, the reverse reaction (DA reaction) can be neglected. The conversion rate of retro DA reaction (X_i) is then determined as the

fraction of formed furfuryl glycidyl ether (FGE), for different times and for different temperatures. It is calculated by the following formula: $Xi = \frac{A_{FGE}}{A_{FGE}+A_{Add}}$

A_{FGE}, A_{Add} are respectively the area of the peak characteristic of one proton in furan group (highlighted in Figure S1a) and the area of a peak representing one proton of the adduct (highlighted in Figure S1c).

The retro DA reaction conversion values of can be found in Table S2.

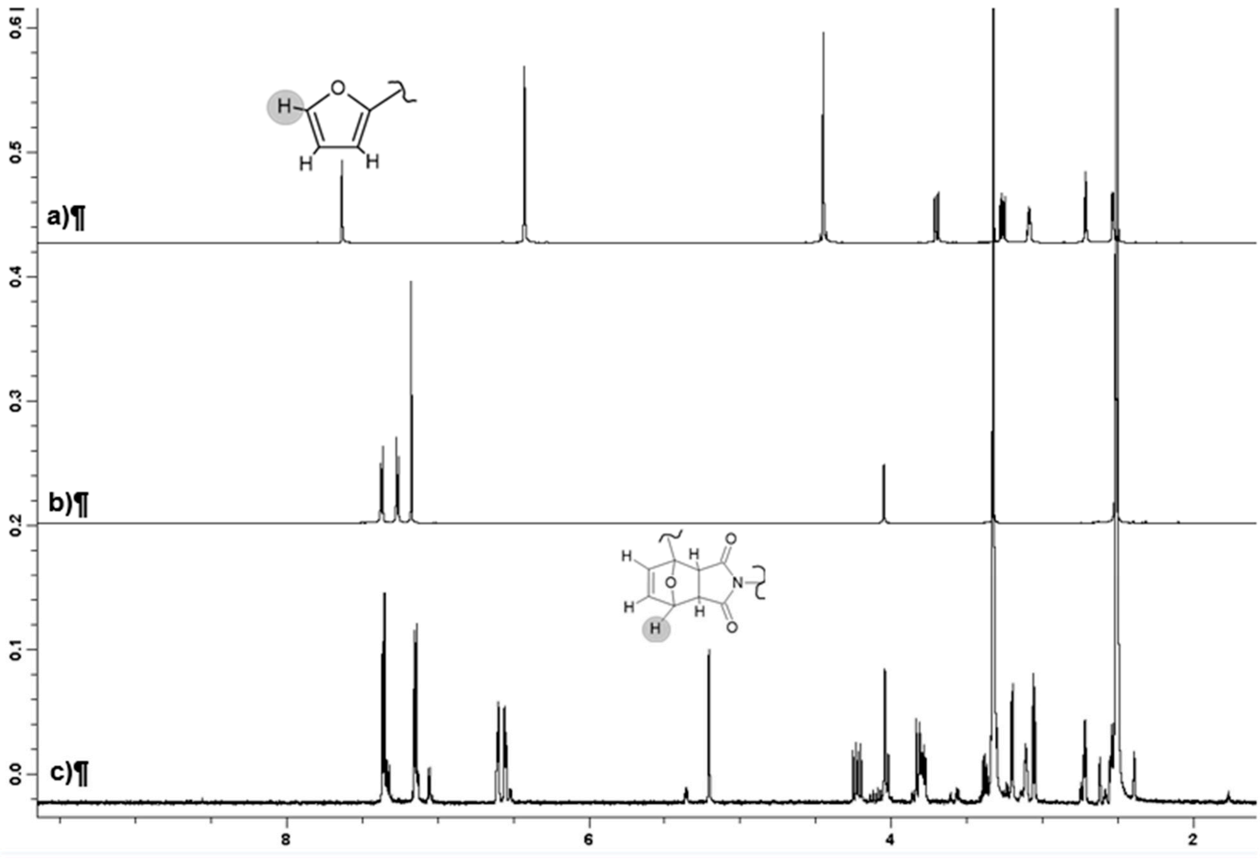

Figure S4. ^{1}H NMR spectra of (**a**) furfuryl glycidyl ether, (**b**) bismaleimide and (**c**) synthesised molecule containing the adduct.

Table S3. Retro DA conversion values determined by ^{1}H NMR spectrometry, for different temperatures.

Time (s)		343 K			353 K	
**0**	0.142	0.029	0.038	0.040	0.028	0.043
**60**	0.155	0.062	0.048	0.167	0.124	0.178
**900**	0.266	0.062	0.048	0.230	0.229	0.231
**1,800**	0.305	0.111	0.075	0.330	0.255	0.309
**2,700**	0.317	0.111	0.090	0.385	0.321	0.412
**3,600**	0.270	0.140	0.147	0.437	0.385	0.398
**7,200**	0.314	0.202	0.253	0.596	0.532	0.664
**10,800**	0.356	0.303	0.315	0.730	0.656	0.773
**18,000**	-	0.384	0.425	0.842	0.839	0.888
**25,200**	-	0.498	0.528	0.900	0.888	0.942

Time (s)		358 K			363 K	
**0**	0.040	0.043	-	0	0.034	0
**60**	0.196	0.199	-	0.285	0.291	0.285
**900**	0.348	0.324	-	0.436	0.463	0.436
**1,800**	0.476	0.473	-	0.559	0.606	0.559
**2,700**	0.579	0.543	-	0.684	0.674	0.684
**3,600**	0.678	0.649	-	0.75	0.773	0.75
**7,200**	0.854	0.837	-	1	0.886	1
**10,800**	0.933	0.919	-	1	0.957	1
**18,000**	0.983	0.983	-	1	0.942	1
**25,200**	1	0.988	-	1	0.955	1

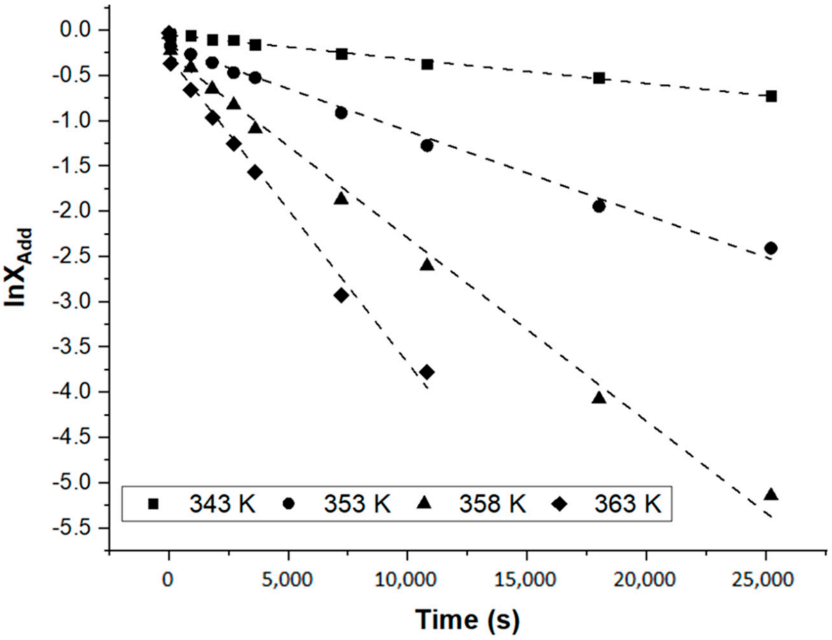

Figure S5. Linearization, according to a first order, of retro DA reaction rate law occurring in the DA molecule in solution performed at different temperatures.
